# Organizational- and system-level characteristics that influence implementation of shared decision-making and strategies to address them — a scoping review

**DOI:** 10.1186/s13012-018-0731-z

**Published:** 2018-03-09

**Authors:** Isabelle Scholl, Allison LaRussa, Pola Hahlweg, Sarah Kobrin, Glyn Elwyn

**Affiliations:** 1 The Dartmouth Institute for Health Policy and Clinical Practice, Dartmouth College, Level 5, Williamson Translational Research Building, One Medical Center Drive, Lebanon, NH 03756 USA; 2 Department of Medical Psychology, University Medical Center Hamburg-Eppendorf, Martinistr. 52, W26, 20246 Hamburg, Germany; 3 Healthcare Delivery Research Program, National Cancer Institute, 9609 Medical Center Drive, Rockville, MD 20852 USA

**Keywords:** Shared decision-making, Decision aids, Implementation, Routine care, Organizational -level characteristics, Health system -level characteristics, Implementation science, Leadership, Incentives, Health policy

## Abstract

**Background:**

Shared decision-making (SDM) is poorly implemented in routine care, despite being promoted by health policies. No reviews have solely focused on an in-depth synthesis of the literature around organizational- and system-level characteristics (i.e., characteristics of healthcare organizations and of healthcare systems) that may affect SDM implementation. A synthesis would allow exploration of interventions to address these characteristics. The study aim was to compile a comprehensive overview of organizational- and system-level characteristics that are likely to influence the implementation of SDM, and to describe strategies to address those characteristics described in the literature.

**Methods:**

We conducted a scoping review using the Arksey and O’Malley framework. The search strategy included an electronic search and a secondary search including gray literature. We included publications reporting on projects that promoted implementation of SDM or other decision support interventions in routine healthcare. We screened titles and abstracts, and assessed full texts for eligibility. We used qualitative thematic analysis to identify organizational- and system-level characteristics.

**Results:**

After screening 7745 records and assessing 354 full texts for eligibility, 48 publications on 32 distinct implementation projects were included. Most projects (*N* = 22) were conducted in the USA. Several organizational-level characteristics were described as influencing the implementation of SDM, including organizational leadership, culture, resources, and priorities, as well as teams and workflows. Described system-level characteristics included policies, clinical guidelines, incentives, culture, education, and licensing. We identified potential strategies to influence the described characteristics, e.g., examples how to facilitate distribution of decision aids in a healthcare institution.

**Conclusions:**

Although infrequently studied, organizational- and system-level characteristics appear to play a role in the failure to implement SDM in routine care. A wide range of characteristics described as supporting and inhibiting implementation were identified. Future studies should assess the impact of these characteristics on SDM implementation more thoroughly, quantify likely interactions, and assess how characteristics might operate across types of systems and areas of healthcare. Organizations that wish to support the adoption of SDM should carefully consider the role of organizational- and system-level characteristics. Implementation and organizational theory could provide useful guidance for how to address facilitators and barriers to change.

**Electronic supplementary material:**

The online version of this article (10.1186/s13012-018-0731-z) contains supplementary material, which is available to authorized users.

## Background

Although recognized as ethically important and frequently included in healthcare policies [1], the practice of engaging patients in their healthcare decisions is infrequently implemented in routine care [2–6]. Research on shared decision-making (SDM) has identified this failure of implementation, but has focused primarily on the associated patient- and provider-level characteristics [7–10]. Studies of other practice-changing interventions have similarly identified implementation challenges, but in other areas, the search for solutions has extended to characteristics of healthcare delivery beyond the patient and clinician to the organizational characteristics and the system-level policies. How these findings from the implementation literature, and research on organizational- and system-level characteristics specifically, might affect efforts to implement SDM is not well known.

SDM is a widely recognized approach to cultivate patient-centered care [11, 12]. It is an approach where clinicians and patients share the best available evidence when faced with the task of making decisions, and where patients are supported to consider options and to achieve informed preferences [13]. SDM is a communicative process that can be supported by the use of decision aids, also called decision support interventions. In the last several years, there has been growing interest in advancing SDM in routine healthcare. In many countries, health policies include implementation of SDM. In a series of articles recently published on the development of activities to promote SDM in 22 different countries, it was shown that 19 countries have health policies that foster or even demand SDM implementation [1]. Despite this health policy commitment to SDM and its inclusion in a range of clinical practice guidelines, study results from other countries point towards poor implementation in routine clinical practice [2–6].

These results have led to work that attempts to explain the difficulty of implementing SDM in routine care. Research on barriers to and facilitators of SDM mostly identifies contributing factors at the individual level of care, i.e., characteristics of individual patients, clinicians, or the direct patient-clinician interaction [8–10]. Two systematic reviews on perceived barriers and facilitators of SDM implementation not only reported individual factors (i.e., knowledge, attitudes, and behavior), but also included a few environmental factors (e.g., time, resources) [10, 14]. A similarly narrow focus on attitudes, skills, and behavior of individual clinicians and patients manifest in most interventions developed for SDM [15]. Recent work has acknowledged the importance of taking organizational-level characteristics into account. These are the characteristics of specific healthcare organizations (i.e., entities that deliver healthcare, e.g., hospitals, practices) that affect the implementation of SDM. For example, Müller and colleagues [16] highlighted the importance of organizational culture, leadership support, and changes in workflow structures to better implement SDM in cancer care. Additionally, little is known about the role of system-level characteristics in the implementation of SDM. These are the characteristics of the healthcare system that guide the work of healthcare organizations (i.e., the political, economic, and social context in which healthcare organizations are embedded, e.g., policies and legislation) [17].

Research on the implementation of health innovations has shown that it is crucial to take into account characteristics of healthcare institutions and of the healthcare system at large in order to change practice [18–20]. Those characteristics may otherwise function as powerful barriers to implementing SDM at the individual encounter level. Nevertheless, implementation strategies are often targeted to change knowledge, attitudes, and behavior of individual providers [21], hindered perhaps, by the lack of measures available to assess system-level characteristics [18]. Similarly, in research on SDM, no studies have focused solely on an in-depth synthesis of the literature around organizational- and system-level characteristics that may influence the implementation of SDM in routine care. A greater understanding of the organizational- and system-level characteristics that could impede or support implementation of SDM in routine care may be helpful in finding ways to address these characteristics in implementation strategies. Thus, the aim of this scoping review is to compile a comprehensive overview of experiences with organizational- and system-level characteristics in implementing SDM in routine care. The following research questions guided this scoping review:

1. What experiences with organizational- and system-level characteristics are reported in SDM implementation projects?
2. What strategies to address these characteristics are discussed in the literature?

## Methods

### Design

We performed a scoping review rather than a systematic review due to the broad nature of our research questions, the young field of SDM research, and our anticipation of high variation in study designs and methodologies [22]. We used the definition of scoping review given by Colquhoun and colleagues: “a form of knowledge synthesis that addresses an exploratory research question aimed at mapping key concepts, types of evidence, and gaps in research related to a defined area or field by systematically searching, selecting, and synthesizing existing knowledge” [23].

### Protocol

We developed our protocol based on the Arksey and O’Malley framework [22], as well as on subsequently published guidance on how to conduct scoping reviews [24–26]. The final version of the protocol can be found in Additional file 1.

### Eligibility criteria

We included publications that reported on the results of projects, quality improvement programs, or studies that aimed to implement SDM, decision aids (i.e., tools for use inside or outside the clinical encounter [27]) or other decision support interventions (i.e., mediated by more interactive or social technologies [27]) in routine healthcare through a certain implementation strategy or effort. To be included, these full texts also needed to report on organizational-level and/or system-level characteristics described to influence the implementation, and/or describe strategies that might address organizational-level and/or system-level characteristics. Opinion pieces, reviews, and study protocols were excluded, but reviews were used in the secondary search process, as described below. The full list of inclusion and exclusion criteria, specifying concepts and contexts of this scoping review, is displayed in Table 1.

**Table 1:** Inclusion and exclusion criteria

Inclusion criteria		Excluded full texts (*N* = 306)
I1	The full text is accessible.	2
I2	Context: the language of the full text is English or German.	0
I3	Concept: the main subject of the full text is shared decision-making (SDM) or decision aids or other decision support interventions.	33
I4	Concept: the full text reports on the results of a project, quality improvement program, or study that aims to implement SDM or decision aids or other decision support interventions in routine healthcare through a certain implementation strategy or effort.	157
I5	Concept: the full text reports on the role of experienced organizational- and/or system-level characteristics that influenced the implementation of SDM, decision aids, or other decision support interventions.	10
Exclusion criteria		
E1	Context: the full text is an opinion piece, commentary, editorial, analysis article, or letter, i.e., does not report on a primary data collection.	61
E2	Context: the full text is a systematic review, a scoping review or a structured literature review.	22
E3	Context: the full text is a study protocol.	21

### Search strategy

We performed an electronic literature search in Medline, CINAHL, and Web of Science Core Collection. We included articles published between January 1997, the year in which Charles and colleagues described the concept of SDM in their seminal article [28], and October 10, 2016. The search was limited to articles published in English or German, as these were the only languages spoken by a minimum of two members of the review team. Details of the search strategies in the different databases can be found in Additional file 2.

Our primary electronic search was complemented by a comprehensive secondary search strategy. All records excluded through criterion E2 (systematic, scoping, and structured literature reviews) [10, 14, 15, 29–42] were checked to see whether they reported on studies that could potentially be relevant for this scoping review. Subsequently, the reference lists of six of these reviews [10, 15, 29, 36, 39] were assessed for eligibility. Furthermore, two books were searched for chapters meeting the inclusion criteria [43, 44], and a gray literature search was conducted on a range of websites listed in Additional file 3.

### Study selection process

We imported all identified records into reference management software (Endnote) and removed duplicates. First, IS and a second reviewer (PH, AL, or RPM) performed an independent title and abstract screening to check for potential inclusion of records. A record was included into the next step of full text assessment if at least one reviewer deemed it appropriate. Second, full text assessment was conducted. To ensure quality and consistency of full text assessments, the first 20% of randomly selected full texts were assessed by two team members (IS and PH or IS and AL). In 83% of the cases, the team members agreed on inclusion or exclusion. Discrepant assessments were subsequently discussed by the team members. This process led to minor revisions in the exact wording of the inclusion and exclusion criteria and an instruction of how to use the criteria. Then, another round of double assessment using another set of 10% of randomly selected full texts was conducted, leading to agreement in 93% of the cases. The subsequent assessment of the remaining 70% of full texts was conducted by one reviewer (IS) using a conservative approach. Whenever the single assessor (IS) was in slight doubt about whether to include or exclude a full text, a second reviewer was assigned to assess that full text, and final decision regarding inclusion was made by discussion. This procedure was done for a total of 14 full texts.

### Data extraction

We extracted general information on each study and specific information related to the research questions. We extracted any information on experiences related to organizational- and system-level characteristics and potential strategies to address them. As we wanted to give a broad overview, we extracted all information on experiences reported in the publications, including experiences derived from results (empirical) and from the interpretation of results (opinion-based). The number of full texts identified and selected is described using the PRISMA flowchart. The initial data extraction sheet was developed by one team member (IS), based on experience from other reviews [12, 15, 45, 46]. It was pilot tested by IS and AL, using two included full texts [47, 48]. We compared the extracted data and found only very minor differences in the level of detail of the respective extractions. As a result, the extraction sheet was slightly revised (e.g., by adding definitions of what to extract). Further data extraction was conducted by one person (either AL or IS). Whenever one data extractor was in doubt regarding what to extract for a certain category, the second person checked the full text and both met to discuss agreement on what to extract.

### Methodological quality appraisal

We did not appraise the methodological quality or risk of bias of the included studies, which is consistent with guidance on the conduct of scoping reviews [22].

### Synthesis

We conducted a descriptive analysis of characteristics of the included studies (e.g., types of study design, years of publication) as well as a qualitative thematic analysis of the organizational- and system-level characteristics identified in the studies. We decided to report what other studies reported as influential characteristics, rather than classify them as barriers or facilitators. This analysis drew on principles of qualitative content analysis described by Hsieh and Shannon [49] and consisted of the following steps: first, two researchers (AL and IS) read the entire set of extracted data to gain an overview. Second, one researcher (AL) coded the material (initial inductive coding). Third, comments by a second researcher (IS) led to adaptation of the coding system. Fourth, the revised codes were organized into a coding system using clusters and subcategories, agreed in discussion with two other team members (GE and SK). Fifth, the material was re-coded by one researcher (AL) using the established coding system. Sixth, the re-coded material was cross-checked by a second researcher (IS) and minimal changes were made in discussion (IS and AL). Potential strategies mentioned in the publications to address organizational- and system- level characteristics were synthesized and mapped onto identified characteristics in a team discussion (IS, AL, GE). No qualitative data analysis software was used. Analyses were conducted on the level of distinct implementation projects, i.e., publications reporting on the same implementation project were grouped under one single project ID.

## Results

### Included studies

**Fig. 1:**
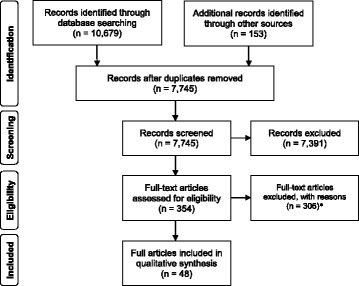
Flow chart of study selection. *Reasons for exclusion: I1: 2 in total (1 full text from primary search, 1 full text from secondary search). I2: none. I3: 33 in total (29 full texts from primary search, 4 full texts from secondary search). I4: 157 in total (113 full texts from primary search, 44 full texts from secondary search). I5: 10 in total (8 full texts from primary search, 2 full texts from secondary search). E1: 61 in total (58 full texts from primary search, 3 full texts from secondary search). E2: 22 in total (17 full texts from primary search, 5 full texts from secondary search). E3: 21 in total (20 full texts from primary search, 1 full texts from secondary search)

After screening 7745 titles and abstracts for eligibility, and checking 354 full texts against the inclusion and exclusion criteria, we included 48 full texts (see Fig. 1). Reasons for exclusion of full texts are displayed in Table 1. The included full texts report on a total of 32 distinct implementation projects. While most projects were only reported in a single publication, several projects were described in two or more publications. Twenty-two projects were conducted in the USA, and 26 projects focused on the implementation of decision aids or other forms of decision support. Projects focused on various settings and a broad range of decisional contexts. Table 2 gives an overview on the included projects and publications.

**Table 2:** Included implementation projects

Project ID	Author (year)	Country	Study design*	Setting	Context	Implemented intervention	Implementation strategy
P1	Abrines-Jaume et al. (2016) [47]	UK	Quality improvement study	Outpatient, inpatient, community, and outreach	Child and adolescent mental health	SDM in general	Teams were encouraged to try a range of tools to support SDM and received cross-site learning events every 3 months including information and materials, group discussions, and action learning sets as part of the Closing the Gap program. They also received regular site meetings and phone and email guidance.
P2	Andrews et al. (2016) [68] Berg et al. (2011) [69] Friedberg et al. (2013) [70]	USA	n/r (descriptive implementation study)	Specialty and primary care in an academic medical center	Orthopedics, breast cancer, hip and knee osteoarthritis, prostate cancer, cancer screening, spine conditions, heart/chronic/other	Decision aids and other form of decision support	When indicated, individual’s treatment preferences, questions, and other decision-making data were shared with their clinician and recorded in their electronic medical record (EMR). Shared decision-making summaries (dashboards) were reported to departments at regular intervals in an effort to systematically monitor and evaluate the use of decision support programs in clinical care.
P3	Arterburn et al. (2016) [71] Conrad et al. (2011) [55] Hsu et al. (2013) [52] Hsu et al. (2013) [72] King and Moulton (2013) [51]	USA	Mixed-methods case study	Specialty care in an integrated health system	Focus on decisions regarding surgical treatments: breast cancer and DCIS, hip and knee osteoarthritis, chronic low back pain, living better with chronic pain, colon cancer screening, depression, diabetes, PSA testing	Decision aids	Senior project management consultants worked with service line leaders to develop implementation agreements and process flow diagrams for each service line. Once a draft distribution process was generated, the project managers met with frontline providers and staff to introduce the DAs, the distribution process and answer questions. Process revisions were based on provider reactions and suggestions. Once an implementation process was agreed upon, a “go-live” date was set, after which the project managers visited each clinic site at least once to monitor implementation processes and progress. Sites experiencing challenges received additional visits and calls as necessary. DAs were distributed using an existing service that supplies educational materials to patients via US mail. The DVD versions of the DAs could be ordered for patients by clinical staff using the electronic health record. Patients could also view the DA online via the patient portal, and providers could embed a link to the video DA in the patient’s after-visit summary. In treatment decision for which the time between a patient’s initial appointment and the procedure was very short, the DAs could also be distributed in the office. Process was monitored using twice-monthly distribution reports given to clinical leaders. In the second year, these reports included more specific numbers for individual clinicians.
P4	Belkora (2011) [73] Belkora et al. (2008) [74] Belkora et al. (2011) [75] Belkora et al. (2012) [48] Belkora et al. (2015) [76]	USA	Quality improvement study	Breast care center (in an NCI designated comprehensive care center)	Breast cancer	Decision aids and other form of decision support	Long-term project with multiple iterations. Implementations consisted of consultation planning, recording, summarizing services in which support staff assisted patients in communicating with their providers before a visit (question brainstorming) and during a visit (audio recording). Improvements on this service consisted of adjusting the scheduling system and workflow of decision support, mailing DAs to patients at home, and making follow-up calls
P5	Belkora et al. 2008 [77]	USA	Post-implementation qualitative study	Community clinics and community resource centers	Breast cancer	Other form of decision support	One-time Consultation Planning training workshops included lectures, structured role playing, and group discussion sessions.
P6	Brackett et al. (2010) [78]	USA	n/r (descriptive implementation study)	Primary care in one academic medical center and one Veteran’s Affairs Medical Center	Prostate cancer and colorectal cancer screening	Decision aids	Four methods were compared: (1) automatic pre-visit mailing to all potentially eligible patients, (2) letter mailed to all potentially eligible patients offering pre-visit DA (3) eligible patients offered DA at checkout from primary care visit (4) clinician prescribes DA to eligible patients during primary care visit
P7	Clay et al. (2013) [79] Friedberg et al. (2013) [70]	USA	n/r (descriptive implementation study)	Academic medical center department of orthopedics	Orthopedics	Decision aids	Embedding decision aid into new EMR to systematically and automatically deliver DA to the right patient at the right time.
P8	Elwyn and Thomson (2013) [80] King et al. (2013) [58] Lloyd et al. (2013) [81] Lloyd and Joseph-Williams (2016) [82]	UK	Service development/quality improvement program	NHS hospitals and primary and secondary care teams	Head and neck cancer, breast cancer, pediatric tonsillectomy, obstetrics, urological problems, ear, nose and throat, knee osteoarthritis, statins, managing mood disorders, sexual health and contraception, upper respiratory tract infection, managing carpal tunnel syndrome, smoking cessation, menorrhagia, long-term care, benign prostatic hyperplasia	SDM in general	Making good decisions in collaboration (MAGIC) improvement program: an approach that integrates shared decision-making into routine care through training in shared decision-making and the use of decision support tools, peer support for clinicians, and support for patients to become more engaged in their care. This program has been implemented at several sites and is adapted for best use in the context of each site.
P9	Elwyn et al. (2012) [83]	UK	Post-implementation mixed-methods study	NHS healthcare professionals	Knee osteoarthritis, amniocentesis, breast cancer, benign prostatic hyperplasia, localized prostate cancer	Decision aids	Tools were made available on NHS Direct’s web platform and patients were directed to tools by staff.
P10	Feibelmann et al. (2011) [84]	USA	n/r (descriptive implementation study)	Cancer centers, hospitals, private practices, and resource centers	Breast cancer	Decision aids	Letters were mailed to providers at sites. Sites could fax or mail back a request for a sample program and then sign a participant agreement to receive copies of decision aids to use with patients. Various implementation techniques were used at individual sites.
P11	Fortnum et al. (2015) [85]	Australia	n/r (descriptive implementation study)	Renal units	End-stage kidney disease	Decision aids	Decision aid PDFs were made available nationally (downloadable from Kidney Health Australia and Kidney Health New Zealand websites). Education was provided to over 2000 ANZ health professionals through teleconferences, webinar, website distribution, state workshops, unit visits, conference presentations, and email.
P12	Frosch et al. (2011) [50] Uy et al. (2014) [86]	USA	n/r (descriptive implementation study)	Primary care offices and community health centers	First prostate and colon cancer screening then expanded to various contexts with 24 different decision aids available	Decision aids	The initial implementation practices received evidence-based brochure decision support interventions (DESIs). The goal was to provide the DESIs to patients at the time of an office visit and to review before the consultation with the physician. In an expansion of this implementation individual practices selected DESIs to provide to patients. Phase 1: during a patient visit, physician or staff would assess appropriateness of DA prescription then eligible patients received package with DA to take home and review before follow up-appointment. The exact logistics of DA distribution were established by practices individually. Weekly “academic detailing” visits were conducted with a member of the research team to identify barriers and develop potential solutions. Phase 2: introduction of a financial incentive to compensate for time spent prescribing DAs and inclusion/exclusion criteria (to ensure that only eligible patients receive the DA) and phone survey instead of questionnaire.
P13	Friedberg et al. (2013) [70] Frosch (2011) [73] Lin et al. (2013) [87] May et al. (2013) [88] Tietbohl et al. (2015) [89]	USA	Case study (descriptive implementation study)	Primary care clinics in an integrated health system	Various contexts: 16 different decision aids available	Decision aids	The project team collaborated with clinics to tailor decision aid distribution methods to individual clinic workflows. Each clinic had a physician and staff champion responsible for promoting the program. The leadership team at each clinic, which included both physicians and leaders of clinical support staff, selected decision aid topics for distribution from the list of available tools. Project team members engaged in academic detailing visits and social marketing efforts to promote distribution of the decision aids.
P14	Garden (2008) [59] Wirrman and Askham (2006) [90]	UK	n/r (descriptive implementation study)	Urology departments	Early localized prostate cancer or benign prostatic hyperplasia	Decision aids	Nurse specialists were trained to implement Decision Support Aids and Decision Quality Assessment Forms to patients (implemented at different points in the care pathway at different sites).
P15	Holmes-Rovner et al. (2000) [91]	USA	Mixed-methods feasibility study	Hospital community health education centers, cardiology education and research departments, and health education libraries	Breast cancer and ischemic heart disease	Decision aids	To ensure local acceptance of the programs and to fit the program into existing routines, hospitals were asked to identify study coordinators who would work with local physicians and nurses to implement the programs. Participating clinicians were asked to review decision aid and complete survey prior to distributing to patients. Clinicians received reminders and study coordinators repeatedly discussed the DAs with them.
P16	Holmes-Rovner et al. (2011) [92]	USA	Retrospective post-then-pre design	Internal medicine and family medicine clinics	Stable coronary artery disease	SDM in general	The complex decision support system called Shared Decision Making Guidance Reminders in Practice (SDM-GRIP) consisted of: (1) provider training (2) patient education. To facilitate discussion in the clinical encounter, a dedicated SDM provider visit was established, and an encounter decision guide (EDG) was given to patients. The EDG provided an evidence summary and decision pages to record choices arrived at in the clinical encounter.
P17	Julian et al. (2011) [93]	USA	n/r (descriptive implementation study)	Comprehensive breast care center	Breast cancer, DCIS	Decision aids and other form of decision support	A nurse navigator coordinated patient care and provided decision aids to women.
P18	Korsen et al. (2011) [73]	USA	n/r (descriptive implementation study)	Primary care in an integrated health system	PSA testing, colorectal cancer screening, diabetes, acute low back pain, chronic low back pain, depression, menopause, advance directives	Decision aids	Implementation included (1) pre-visit, visit-based, and post-visit distribution models, (2) use of EHR for DA referral, (3) various trainings, workshops, and presentations at different sites
P19	Friedberg et al. (2013) [70] Lewis et al. (2011) [73] Lewis et al. (2013) [57] Miller et al. (2012) [94]	USA	n/r (descriptive implementation study)	Primary care clinic	PSA testing and weight loss surgery	Decision aids	The focus was on automated DVD DA delivery through EHR and social marketing campaign. Five delivery models were used: (1) mailing DAs prior to visit (2) using Patient Health Survey to identify eligible patients and allow them to request a DA, (3) requesting DAs by physician (4) distributing DAs within chronic disease management program (5) pre-visit online screening for DA eligibility
P20	McGrail et al. (2016) [95]	USA	n/r (descriptive implementation study)	One primary care clinic, one general hospital	Statins, anticoagulation in patients with atrial fibrillation, osteoporosis and knee osteoarthritis, urinary incontinence	SDM in general	The SHARE approach “train-the-trainer” workshop was followed by training sessions for residents and medical group staff.
P21	Mollicone et al. (2013) [96]	USA	n/r (descriptive implementation study)	Specialty care center	Chronic kidney disease	SDM in general	Treatment Options Program (TOPs) consists of free classes offered locally, nationwide, by trained FMCNA personnel to educate patients and family members about the options for treatment. Follow up calls encourage patients to discuss options with their doctors and participate in their care.
P22	Friedberg et al. (2013) [70] Morrissey and Elwyn (2013) [97] Morrissey and Michels (2011) [98]	USA	n/r (descriptive implementation study)	Primary care	Benign prostatic hyperplasia, prostate cancer, breast cancer, depression, uterine fibroids, chronic low back pain, chronic pain, menopause	Decision aids and other form of decision support	Three models for implementation were used: (1) patient referred from primary care or specialist for care coordination/navigation which included face to face visit with DA (2) provider teed up SDM conversation in exam room and handed patient off to nurse who provided information and DA (3) patient requested DA and care coordinator follows up with a call for discussion
P23	Newsome et al. (2012) [60]	USA	Post-implementation qualitative study	Family medicine clinics	Cancer screening, chronic illness care	Decision aids	Physicians used the DAs in clinical practice and medical assistants were involved in distribution of DAs (details not specified, reported in a separate publication).
P24	Pasternack et al. (2011) [99]	Finland	n/r (descriptive implementation study)	Breast cancer screening providers	Breast cancer screening	Decision aids	Letter templates with invitation to screening and short decision aid on the back where made available to all breast cancer screening facilities and municipalities in the country. The short DA was put on the back of the letter to avoid extra costs for the providers, who usually just send out the invitation. A website contained a more in depth decision aid. The service providers received information on legislation, the new letter templates, and posters for the waiting rooms.
P25	Sepucha and Simmons (2011) [73] Sepucha et al. (2016) [100] Simmons et al. (2016) [101]	USA	n/r (descriptive implementation study)	Primary care clinics	Various contexts: 40 different decision aids available	Decision aids	Clinicians were able to order DAs through the electronic medical record (EMR). The EMR application then generated a note in the patient’s chart documenting that the material has been sent. The distribution and inventory of DA were managed centrally. The DAs were available in several formats (e-mail message with a link to access the DA online; DVD and booklet in the mail). Early on DA prescription was done in a visit by the clinician, but the SDM implementation team worked with clinicians and administrators to automatize prescriptions. Some years into the implementation program, a short 1 h training module was delivered to clinicians to increase familiarity with the DAs, show them ordering in EMR and discuss implementation challenges. They received CME points for training. Further into the implementation program, patients received the opportunity to order DAs themselves (patient-directed ordering). There were no mandates or long-term financial incentives or penalties associated with using or not using DAs
P26	Silvia et al. (2008) [102] Silvia and Sepucha (2006) [103]	USA	Post-implementation qualitative study	Community resource centers, community hospitals, academic centers, community oncology center	Breast cancer	Decision aids	Providers and resource centers across the country were informed about the availability of the programs through letters and e-mail. Interested sites received free copies and were left to decide themselves how to use them.
P27	Stacey et al. (2006) [104]	Canada	n/r (descriptive implementation study)	Call center	Various health issues; birth control methods, breast versus bottle feeding, male newborn circumcision, wisdom teeth removal, and treatment of miscarriage most common	Decision aids and other form of decision support	Interventions included an online auto tutorial, skill-building workshop, decision support protocol, and feedback on quality of decision support provided to simulated callers
P28	Stacey et al. (2008) [105]	Australia	Pre-post test study	Cancer call center	Cancer	Other form of decision support	Interventions included a decision support tutorial, skill-building workshop, and decision coaching protocol. Supervisors were trained in decision support, a trainer workshop was held for supervisory staff members, and the director of the cancer helpline addressed workshop participants to validate that decision support is an important part of their call center role.
P29	Stacey et al. (2015) [106]	Canada	Prospective pragmatic observational trial	Cystic fibrosis clinics	Adults with cystic fibrosis considering referral for lung transplant	Decision aids and other form of decision support	Implementation strategy was based on results of prior barriers survey. It consisted of training (workshop and online tutorial), easy access to decision aids, and conference calls for ongoing support. Patients completed DA on their own and discussed results with provider at a subsequent encounter, and a summary was included in the clinic record.
P30	Stapleton et al. (2002) [107]	UK	Post-implementation qualitative study	Women’s homes, maternity clinics	Antenatal care and maternity services	Decision aids	Leaflets were provided as part of a cluster randomized controlled trial. Health professionals received a training session in how to use them.
P31	Swieskowski (2011) [73]	USA	n/r (descriptive implementation study)	Primary care clinics	Acute and chronic low back pain, diabetes, women’s health issues, knee and hip osteoarthritis, cardiac conditions, spinal care, end of life care, PSA testing	Decision aids	Potential patients were identified by pre-visit chart review and DAs were prescribed by providers or health coaches during the visit. Follow-up decision support was provided by the physician or the health coach at a follow-up visit.
P32	Tapp et al. (2014) [53]	USA	Process improvement study	Primary care practices	Asthma	SDM in general	A community based participatory research approach was used to form an advisory board (including patients, physician champions, other healthcare professionals, administrative staff) that met monthly to tailor intervention to needs of each practice (e.g., adapting intervention to delivery by different types of staff members, adapting material for use by Spanish-speaking, low literacy and pediatric population, decide on roll out schedule). All practices started with kick-off meeting, then discussion rounds around logistics, training sessions (including use of decision support materials), regular follow-up meetings.

*UK* United Kingdom, *USA* United States of America, *SDM* shared decision-making, *DCIS* ductal carcinoma in situ, *PSA* prostate-specific antigen, *NCI* National Cancer Institute, *NHS* National Health Service,

*Study design: as reported in publication; if not reported (n/r), authors categorized based on study description in brackets

### Characteristics influencing SDM implementation

**Fig. 2:**
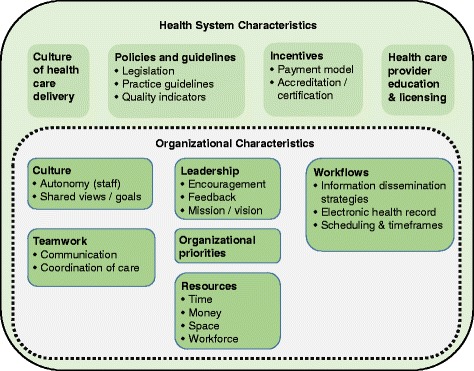
Overview of identified characteristics. Main categories are displayed in bold; subcategories are listed as bullet points. The dashed line around the organizational characteristics indicates that these characteristics are influenced by health system characteristics

Figure 2 gives an overview of the identified characteristics.

#### Organizational-level characteristics

Table 3 displays the organizational-level characteristics reported in the included full texts as influencing the implementation of SDM, decision aids, or other decision support interventions. The table includes descriptions of all identified characteristics. Six main categories of organizational characteristics were described in the included studies: organizational leadership, culture, teamwork, resources, priorities, and workflows. Five of the six main categories also included several subcategories of organizational-level characteristics; for example, the category “organizational resources” included the subcategories time (that healthcare providers have per patient), financial resources (that are available for certain activities within a healthcare organization), workforce (i.e., employees available for and assigned to certain activities within a healthcare organization), and space (i.e., room available for certain activities within a healthcare organization). Both the availability of resources within an organization and organizational workflows (e.g., patient information dissemination strategies, scheduling routines, use of the electronic health record) were described to have influenced SDM implementation efforts in over three quarters of the projects, and facets of the organizational culture and teamwork within an organization were reported in only a third of the projects (see column “Project IDs” in Table 3).

**Table 3:** Identified organizational-level characteristics

Characteristics	Descriptions^#^	Project IDs*
Organizational leadership		
2003 corporate mission and vision statement	Degree to which the description of the organization’s core purpose and vision for the future supports SDM	P3, P8, P13, P25, P27, P28
Encouragement	Degree to which leaders in organization proactively support SDM	P1, P2, P3, P4, P5, P8, P12, P13, P14, P25, P26, P27, P31
Performance measurement and feedback	Use of results of performance measurement or quality indicator metrics to indicate room for improvement	P2, P3, P7, P8, P13, P16, P18, P25, P27, P28, P32
Organizational culture	Degree to which an organization’s culture supports SDM	P2, P3, P8, P12, P13, S14
Autonomy of staff	Degree of flexibility that healthcare providers (HCPs) have to achieve organizational goals	P1, P3, P8, P26
Shared views and goals	Degree to which team members share the same views and goals	P4, P8, P9, P13, P21, P31
Organizational teamwork		
Communication	How information is shared within and between teams	P7, P8, P12, P13, P22, P32
Coordination of care	Deliberate organization of care by HCPs from different specialties	P3, P7, P12, P13, P14, P16, P26, P32
Organizational resources	Availability of resources	P12, P22, P26
Time	Amount of time HCPs have per patient/patient visit	P1, P3, P5, P8, P9, P10, P12, P13, P14, P15, P19, P26, P27, P28, P29, P30, P31, P32
Financial resources	Amount of money available for certain activities within organization	P2, P3, P4, P5, P11, P14, P19, P31
Space	Amount of room available for certain activities within organization	P4, P5, P8, P26
Workforce	Availability and assignment of employees for certain activities within organization	P3, P4, P5, P8, P10, P12, P14, P18, P19, P22, P23, P27, P31, P32
Organizational priorities	Degree to which other aspects of care delivery conflict or align with SDM	P2, P3, P4, P5, P8, P9, P10, P12, P13, P14, P18, P19, P26, P27, P31
Organizational workflows		
Patient information dissemination strategies	Availability of methods to disseminate information to patients and compatibility of workflows with decision aid distribution processes	P2, P3, P4, P5, P6, P8, P12, P13, P14, P17, P22, P24, P25, P26, P27, P28, P29, P31
Scheduling routines and timeframes	Degree to which scheduling (e.g., of appointments or for procedures) and time frame available until decision is needed impacts SDM	P3, P4, P6, P8, P10, P12, P13, P14, P15, P21, P22, P26, P29
Electronic health record (EHR)	Availability of an EHR to be used in SDM (e.g., documentation of process)	P2, P3, P6, P7, P8, P13, P14, P17, P18, P19, P20, P23, P27, P31

*HCPs* Healthcare providers, *EHR* electronic health record, *SDM* shared decision-making

^#^The descriptions are the result of the thematic analysis

^*^For projects described in more than one publication, at least one publication had to report on a specific characteristic to be listed in this table

#### System-level characteristics

While many organizational characteristics were identified in the included full texts, only four main categories of characteristics of the healthcare system were described: incentives (i.e., the role of payment models and accreditation/certification criteria), policies and guidelines (i.e. the role of healthcare legislation and clinical practice guidelines), culture of healthcare delivery, and healthcare provider education and licensing. Table 4 gives an overview of the characteristics of the healthcare system that were reported as influencing implementation. The table includes descriptions of all identified characteristics. While only four projects reported that the culture of healthcare delivery influenced SDM implementation, about one third of the projects reported that incentives, policies and guidelines, and healthcare professional education and licensing influenced SDM implementation (see column “Project IDs” in Table 4).

**Table 4:** Identified system-level characteristics

Characteristics	Descriptions^#^	Project IDs*
Incentives		
Payment model	Impact of payment models on the use of SDM	P2, P3, P8, P13, P15, P16, P21, P26, P31, P32
Accreditation/certification criteria	Degree to which SDM is included as a criterion in accreditation/certification standards for healthcare institutions	P3
Policies and guidelines		
Legislation	Degree to which state or national legislation requires the use of SDM/decision support	P3, P14, P19, P21, P29
Practice guidelines	Degree to which relevant practice guidelines support the use of SDM	P2, P3, P9, P26, P27, P28
Quality indicators	Degree to which quality indicators support the use of SDM	P3, P8, P13, P15
Culture of healthcare delivery	Degree to which the culture of healthcare delivery supports SDM	P13, P14, P16, P22
HCP education and licensing	Degree to which HCP initial and continuing education and licensing includes SDM training	P3, P8, P10, P13, P14, P16, P23, P25, P26, P31

*HCPs* healthcare providers, *SDM* shared decision-making

^#^The descriptions are the result of the thematic analysis

*For projects described in more than one publication, at least one publication had to report on a specific characteristic to be listed in this table

### Strategies to address organizational- and system-level characteristics

A range of possible strategies to address organizational- and system-level characteristics and thereby potentially foster SDM implementation were discussed in the publications and mapped onto the identified characteristics. They are displayed in Table 5. Similar to the results on experienced characteristics, most proposed strategies focused on the organizational level. Most studies identified workflow as an organizational-level characteristic influencing SDM implementation and also generated potential strategies to tackle that characteristic. Few strategies were suggested to change organizational culture [50–52], which was also described in fewer studies. A large range of potential strategies were also described to promote leadership activities that might facilitate SDM implementation (see full list in Table 5). At the system-level, fewer strategies were described. Suggestions included changes in payment models [53–55], legislation [51, 56, 57], and health professional education [51, 58–60].

**Table 5:** Described strategies to address identified characteristics

Characteristics	Strategies described
Organizational-level strategies	
Organizational leadership	
Corporate mission and vision statement	Develop and promote a strong consistent message about importance of SDM [72] Make the value of SDM clear to physicians [83] Revise policy and procedure documents to include SDM in those directives [104, 105]
Encouragement	Appoint an internal champion/have clinical champions [7, 54, 58, 59, 68, 87, 100, 103, 108] Provide personal testimonials from leaders [51] Support healthcare professionals (HCPs) in learning SDM skills, e.g., by protecting time to get trained [7, 47, 51, 58] Support SDM implementation at all levels of the organization’s leadership [51, 59, 100, 102] Show interest by doing site visits to clinics/teams implementing SDM [7] Share success stories in grand rounds [58]
Performance measurement and feedback	Provide continuous performance monitoring and feedback on SDM performance, decision aid distribution rate, decision quality, and patient satisfaction rates [7, 52, 53, 58, 69, 72, 81, 92, 104, 105, 108, 109]
Organizational culture	Foster a well-organized and amicable work environment [50] Align SDM implementation with organization’s existing patient-centered philosophy and quality improvement spirit [51, 52]
Autonomy of staff	Allow flexible use of decision aids and freedom on how to achieve SDM implementation goals [7, 47, 51]
Shared views and goals	Address relational dynamics of healthcare teams before SDM implementation [89] Hold regular meeting to share goals and successes [54]
Organizational teamwork	
Communication	Foster frequent, timely, accurate, and problem solving communication about SDM implementation within and between teams [7, 89, 97]
Coordination of care	Implement multidisciplinary teams [79, 102] Have a patient navigator [102] Have a clear definition of team members’ roles [50, 53]
Organizational resources	
Time	Decrease pressure for short patient interactions [105]/expand time to spend with patient [58, 103] Tailor interaction length guidelines for type of interaction [104]
Financial resources	Obtain funding for SDM activities [90] Have access to high quality decision aids at low or no cost [52]
Space	Use offices instead of clinical exam rooms for delivering decision support [74]
Workforce	Engage non-physician personnel (e.g., nurses, office staff) [60, 70, 73, 90] Use unpaid or paid student interns or volunteers to deliver decision support [76, 77] Reorganize workforce responsibilities from over utilized to underutilized staff [74] Fund/hire a decision support/ care coordinator [77, 98] Salaried physicians for which SDM is part of employment obligations [51]
Organizational priorities	Integrate SDM into other interventions or changes (e.g., health coaching, chronic disease management program) [7, 94, 110] Align SDM with wider objectives of the organization (e.g., quality and safety) [7, 58]
Organizational workflows	
Patient information dissemination strategies	Automate decision aid distribution, e.g., pre-visit [78], based on triggers [70], send by mail [58, 75, 90] Keep decision aids/tools accessible in exam rooms and workspaces [7, 86, 87] and make them easily available electronically [7, 58, 105] Offer in-office viewing of decision aids as well as other options (e.g., lending them to patients) [52] Align delivery of decision aids with other aspects of care (e.g., obtaining informed consent) [91] Partner with resource centers to deliver decision support [77] Clarify the place that decision aids have in the clinical pathway [103] Make decision aids available via a state-run website [51] Create protocols to prompted staff members to prescribe decision aid corresponding to the reason for referral [70]
Scheduling routines and time frames	Get decision aids to patients prior to consultations [50, 52] Install scheduling system for SDM/decision aids/decision support [74, 103, 108] Require slowing down the flow of decision-making/reduce time pressure on patient path to treatment decision [58, 91] Allow for flexible patient pathways and scheduling [7, 75]
Electronic health record (EHR)	Use EHR to prompt and document SDM process [7, 54, 70, 73] Use EHR (and merge it with computerized scheduling data) to identify patients eligible for decision aids [69, 73, 78, 87, 90] Have decision aids available on EHR for easy access and have them available of patient portal on EHR [52, 58, 95, 104, 108]
System-level strategies	
Incentives	
Payment model	Use a payment model that motivates providers to engage in SDM (e.g., patient-centered medical home) [51, 52, 92] Reimburse the use of a decision aid and time spent engaging in SDM conversation [91, 96, 103] Move away from fee-for-service to alternative model (e.g., pay-for-performance) [53–55]
Accreditation/certification criteria	Revise accreditation/certification criteria by adding the implementation of SDM as criterion/quality indicator [51]
Policies and guidelines	
Legislation	Create state legislation that fosters SDM (e.g., comparable to Washington state: enhanced legal protection when doing SDM) [51, 56, 57] Create legislation that encourages healthcare organization structures that support SDM [51]
Practice guidelines	Incorporate the use of SDM in clinical practice guidelines [103, 105]
Quality indicators	Make the use of decision aids a quality of care indicator/list SDM as performance metric [55, 87, 91] Health plans could collect and distribute SDM performance data [51] Use a national set of measures [58]
Culture of healthcare delivery	Promote culture of patient engagement in medical school [59]
Education and licensing	Incorporate SDM communication skills (as compulsory) into medical school and residency curricula, as well as into state medical licensing criteria [51, 58–60] Offer CME/CEU credits for watching decision aids/for SDM training [54, 84, 109]

*HCPs* healthcare providers, *EHR* electronic health record, *SDM* shared decision-making, *CME* continuing medical examination, *CEU* continuing education units

## Discussion

### Summary of the review findings

We described a broad range of organizational- and system-level characteristics that were experienced as influencing the implementation of SDM in routine care, as well as strategies to potentially address those characteristics. Included studies reported more often on characteristics influencing the organizational level than the health system level. The reported organizational characteristics are strongly influenced by health system characteristics; for example, the amount of time that a HCP has for a patient’s visit is linked to payment models, the organizational culture is influenced by the general culture of healthcare delivery, and the leadership decisions within an organization are affected by policies, payment models, and accreditation criteria. As the identified characteristics can be barriers, facilitators or both barriers and facilitators to SDM implementation, we described them in a value-neutral way.

### Strengths and limitations

We extracted reports from implementation studies described in any part of the included publications. Our analyses therefore cannot differentiate between experiences based on results and those reflecting interpretation of results. However, for a young research field, we believe this broad scoping review is an important first step to gaining an overview of the topic.

A second limitation is that the primary search was limited to three electronic databases, so we might have missed relevant publications. However, we prioritized sensitivity in our electronic search, which is reflected by the high number of screened abstracts, to identify most relevant work. Furthermore, we conducted an extensive secondary search, including gray literature to find more work not indexed in the electronic databases searched. Another limitation is that we did not conduct a full double assessment and double data extraction. However, we did our best to minimize error by consulting with a second reviewer whenever there was the slightest doubt. A main strength of this review is that it is the first of its kind to focus solely on the impact of organizational and system characteristics on the implementation of SDM. In previous work, the focus had mainly been on the individual clinician-patient level, and organizational- and system-level characteristics had not been examined in depth [10, 14]. Furthermore, it was conducted in an inter-professional and international team.

### Comparison to previous work

First, these findings need to be compared to previous work on SDM. Our results reinforce prior calls for better coordination of care, engagement of non-physician personnel, and the use of the electronic health record (EHR) to implement SDM in previous work [61]. The suggestions to use clinical practice guidelines, postgraduate training, and accreditation as means to better implement SDM [5] are also reflected in the data collected in this scoping review. Many of the characteristics identified in this review have been discussed in trials of SDM interventions or decision aids, in studies of clinicians’ perceptions, or in opinion pieces, but this is the first piece of work looking at characteristics experienced in actual implementation studies.

Second, the results need to be compared to more general work in healthcare implementation science, beyond the case of SDM as a particular innovation to implement. Implementation frameworks and conceptual models like the one postulated by Greenhalgh and colleagues [20] or the Consolidated Framework for Implementation Research (CFIR) [19] have described elements in the inner and outer settings to influence implementation. Our results found a range of very similar characteristics on the organizational level to the ones described in the inner setting, e.g., communication and culture within an organization, leadership engagement, resources, and priorities. However, some of the characteristics we found (e.g., workflows) were not described in the CFIR [19]. One could hypothesize that these aspects are more focused around decision aid implementation and therefore not included in a more general implementation framework. Similarly, several of our system-level characteristics map well onto the CFIR’s outer setting (i.e., aspects around policies, guidelines, and incentives), but the culture of the healthcare system and education and licensing of healthcare professionals cannot be found in the framework [19]. Furthermore, a systematic review on determinants of implementation of preventive interventions on patient handling identified a total of 45 environmental barriers and facilitators [62] that overlap with experienced organizational characteristics identified in our scoping review, particularly the availability of resources, leadership support, and the organization of workflows. Overall, our results in the field of SDM display many similarities with the characteristics described in implementation science frameworks and in other fields of health innovation. However, as we also identify characteristics less described in implementation science literature, we believe it is important to not to be restricted by such frameworks, but enrich them with derived empirical evidence.

Third, some of the strategies recommended by the included projects to intervene on characteristics influencing SDM implementation (Table 5) are vague and require further specification and tailoring to a specific context [63]. For example, some of the strategies that fall into the leadership category could benefit from distinguishing which level of leadership should take action for which strategy. While the people in a governing board of an organization might be the ones to revise mission statements, executive leadership, and departmental management might be the ones who create a culture that supports SDM [64]. Furthermore, all other categories identified as organizational-level characteristics, despite not specifying who should be in charge of making specific changes, imply that organizational leadership is the actor here. Although it is not specified, for example, who should implement multidisciplinary teams or create an SDM coordinator position, there is an implicit assumption that these are leadership tasks. Beyond looking at implementation literature, it might therefore be worthwhile for stakeholders working on SDM implementation to look into organizational theories in healthcare [65], e.g., on the effective organization of healthcare teams or on strategies to restructure healthcare organizations.

### Implications and suggestions for further work

As healthcare systems are complex and composed of components that act nonlinearly [66], a certain identified characteristic can be a facilitator to one stakeholder and a barrier to another. Therefore, more work is needed to move beyond the descriptive stage of this review, especially as differences in the numbers of studies reporting on certain characteristics do not necessarily mean that those characteristics are the most important. Similarly to Koppelaar et al. [62], we believe there is a need to quantify the influence of the identified characteristics, especially as this scoping review’s broad nature is not distinguishing between experiences based on results of implementation studies and interpretation of those results. By evaluating the influencing characteristics in implementation studies, we could analyze interactions between characteristics and find out which of them predict implementation outcomes [18].

As the included studies were predominantly from the USA, future work needs to assess the importance of the identified characteristics in different healthcare systems with variation in financing, coverage, spending, utilization, capacity, and performance [67], as well as different fields of healthcare (e.g., cancer care, mental healthcare). This would help to gain a more specific insight that could foster prioritization of the most important characteristics in a particular setting and strategies to address them.

## Conclusion

Although infrequently studied, organizational- and system-level characteristics appear to play a role in the failure to implement SDM in routine care. A wide range of characteristics described as supporting and inhibiting implementation were identified. Future studies should quantify these characteristics’ differential impact on SDM implementation, their likely interactions, and how different characteristics might operate across types of healthcare systems and areas of healthcare. Healthcare organizations that wish to support the adoption of SDM should carefully consider the role of organizational- and system-level characteristics. Implementation and organizational theory could provide useful guidance for how to address facilitators and barriers to change.

## Additional files

Additional file 1: Protocol. (PDF 129 kb)

Additional file 2: Electronic searches. (PDF 24 kb)

Additional file 3: Gray literature search. (XLSX 15 kb)

## Abbreviations

CFIR: Consolidated Framework for Implementation Research
EHR: Electronic health record
RCTs: Randomized controlled trials
SDM: Shared decision-making
