# Supplementary material for: Organizational- and system-level characteristics that influence implementation of shared decision-making and strategies to address them — a scoping review
Source: Implement Sci. 2018 Mar 9;13:40. doi: 10.1186/s13012-018-0731-z (PMC5845212; doi:10.1186/s13012-018-0731-z)
Supplement: Supplementary file 1 — Protocol. (PDF 129 kb) [file 13012_2018_731_MOESM1_ESM.pdf]

Organizational- and system-level factors that influence the implementation of SDM – a scoping review protocol

Isabelle Scholl[1], Allison LaRussa[2], Pola Hahlweg[3], Sarah Kobrin[4], Glyn Elwyn[5]

Affiliations & email addresses:
1 Dartmouth College, The Dartmouth Institute for Health Policy & Clinical Practice, Isabelle.Scholl@Dartmouth.edu
2 Dartmouth College, The Dartmouth Institute for Health Policy & Clinical Practice, Allison.Larussa.GR@dartmouth.edu
3 University medical Center Hamburg-Eppendorf, Department of Medical Psychology, p.hahlweg@uke.de
4 National Cancer Institute, Healthcare Delivery Research Program, Division of Cancer Control and Population Sciences, kobrins@mail.nih.gov
5 Dartmouth College, The Dartmouth Institute for Health Policy & Clinical Practice, glynelwyn@gmail.com

Corresponding author: *Isabelle Scholl, The Dartmouth Institute for Health Policy & Clinical Practice, Level 5, Williamson Translational Research Building, One Medical Center Drive Lebanon, NH 03756*

Funding: *This work is supported by a Harkness Fellowship in Healthcare Policy and Practice awarded to Isabelle Scholl by the Commonwealth Fund and B. Braun Stiftung. This protocol was developed independently from the funders.*

Registration: *This protocol is not registered.*

Note: *This protocol adheres to the PRISMA-P checklist for the development of review protocols* (Moher et al., 2015)*, as far as applicable to the scoping review methodology. Currently, there is no reporting guidance for scoping reviews, however this seems to be forthcoming (PRISMA-*ScR) (Colquhoun et al., 2014). Upon *completion of the scoping review, the website of the Equator Network will be checked again to determine the availability of this guidance document.*

Introduction

In the last years, there has been growing interest in advancing shared decision-making (SDM) in routine healthcare. In many countries, health policy demands the implementation of SDM. However, despite this legal commitment towards SDM and its inclusion in a range of clinical practice guidelines, it seems poorly implemented in routine care (Charles, Gafni, & Whelan, 2004; Coulter, 2009; Foundation for Informed Medical Decision Making, 2009; Stiggelbout et al., 2012; Vogel, Helmes, & Hasenburg, 2008).

A lot of work on barriers and facilitators of SDM identified mostly contributors at the individual level of care (Légaré, Ratté, Gravel, & Graham, 2008; Shepherd, Tattersall, & Butow, 2007, 2008). More recent work, has acknowledged the importance to take the organizational level (e.g. organizational culture, innovation climate, leadership effectiveness, inter-professional collaboration, clinical information systems, quality management documents) into account (Müller, Hahlweg, & Scholl, 2016). This is in line with research on the implementation of health innovations, which has shown that it is crucial to take into account variables at the level of health institutions in order to change practice (Chaudoir, Dugan, & Barr, 2013; Damschroder et al., 2009). These institutional characteristics will otherwise function as powerful barriers towards implementing SDM at the individual encounter level.

To date, no studies have synthesized the literature around organizational- and system-level factors that influence the implementation of SDM in routine care. However, this would be an important step in order to be able to explore solutions that address these factors. Thus, the aim of this scoping review is to compile a comprehensive overview on organizational- and system-level factors that influence the implementation of SDM in routine care.

Methods

We use the definition of scoping review given by Colquhoun and colleagues, describing it as "a form of knowledge synthesis that addresses an exploratory research question aimed at mapping key concepts, types of evidence, and gaps in research related to a defined area or field by systematically searching, selecting, and synthesizing existing knowledge"(Colquhoun et al., 2014).

We base our methodology on the Arksey and O'Malley framework (Arksey & O`Malley, 2005), as well as on subsequently published guidance on how to conduct scoping reviews (Daudt, van Mossel, & Scott, 2013; Khalil et al., 2016; Levac, Colquhoun, & O'Brien, 2010). The following six steps will be conducted and are described below: 1) identifying the research question, 2) identifying relevant studies, 3) study selection, 4) charting the data, 5) collating, summarizing and reporting the results. We will not appraise the methodological quality or risk of bias of the included studies, which is consistent with guidance on the conduct of scoping reviews (Arksey & O`Malley, 2005).

1) Identifying the research question

In this first step of the scoping review, its objective, purpose, envisioned outcome, and review questions are specified and the concepts in the review questions are defined.

This step has already been conducted, leading to the following specifications.

Objective: *to compile a comprehensive overview on organizational- and system-level factors that have shown to influence the implementation of SDM in routine care.*

Purpose*: A greater understanding of the organizational- and system-level factors that influence implementation of SDM in routine care, may be helpful to find ways to acknowledge these factors in implementation strategies in order to foster the implementation of SDM in routine care.*

Envisioned outcome: *a list of main organizational- and system-level characteristics and proposed solutions to address these characteristics towards the implementation of SDM in routine care.*

Review questions (RQ)
RQ 1: What is known from the existing literature about organizational- and system-level barriers and facilitators towards the implementation of SDM in routine care?
RQ 2: What solutions that address these characteristics are discussed in the literature?
Definition of the concepts in the RQs:

- organizational-level factors: characteristics of a healthcare organization or institution (e.g. a hospital, a practice); also described as institutional level or meso level of care
- system-level factors: characteristics of the health care system, i.e. factors that influence more different organizations
- SDM: 'an approach where clinicians and patients share the best available evidence when faced with the task of making decisions, and where patients are supported to consider options, to achieve informed preferences' (Elwyn et al., 2010). While SDM is a communicative process that can be achieved through the use of decision aids, decision aids are not necessary nor sufficient for SDM (Elwyn et al., 2016).
- Implementation: putting something (e.g. an intervention, in this case SDM) into action in routine health care

2) Identifying relevant studies
The following electronic databases will be searched: Medline, CINAHL, and Web of Science Core Collection. We will limit the search to articles published after 1997, the year in which Charles and colleagues described the concept of SDM in their seminal article (Charles, Gafni, & Whelan, 1997). The search will also be limited to articles published in English or German. The secondary search strategy will consist of reference tracking of included articles and contacting of experts. Grey literature will be included by searches on a range of websites (see separate document).

3) Study selection
This scoping review includes primary and secondary studies that report on empirical data regarding organizational- and system-level factors that influenced the implementation of SDM in routine care. Final eligibility (inclusion and exclusion) criteria will be developed post hoc, based on increased familiarity with the literature.

This stage will be considered an iterative process involving searching the literature, refining the search strategy, and reviewing articles for study inclusion.
Two researchers will then use those criteria to independently screen the titles and abstracts of the retrieved records and assess eligibility of full-texts. Disagreements about study inclusion will be resolved by discussion. If necessary, a third reviewer will be consulted. The study selection process will be supported by importing all identified records to a reference management software and by assigning them individual ID numbers.

4) Charting the data

The data charting form will be developed by one team member, then pilot tested by two team members using two included studies, and subsequently revised to reach consensus on the data collection process and to add additional categories. As this step is considered an iterative process, the charting form will be continually updated if necessary. We will both extract general information on each study and specific information related to RQs.
The search decision process will be described narratively as well as using the PRISMA flowchart.

5) Collating, summarizing and reporting the results

We will conduct a descriptive numerical analysis of characteristics of the included studies (e.g. types of study design, years of publication), as well as a qualitative thematic analysis of the organizational- and system-level factors identified in the studies. The results of this thematic analysis will be reported in a tabular form (i.e. a list of factors). Furthermore, the meaning of the results will be discussed in relation to the overall study purpose.

References

Arksey, H., & O`Malley, L. (2005). Scoping studies: Towards a methodological framework. *International Journal of Social Research Methodology*, *8*(1), 19–32. Journal Article.

Charles, C., Gafni, A., & Whelan, T. (1997). Shared decision-making in the medical encounter: What does it mean? (or it takes at least two to tango). *Social Science and Medicine*, *44*(5), 681–692. Journal Article. http://doi.org/10.1016/S0277-9536(96)00221-3

Charles, C., Gafni, A., & Whelan, T. (2004). Self-reported use of shared decision-making among breast cancer specialists and perceived barriers and facilitators to implementing this approach. *Health Expectations*, *7*(4), 338–348. Journal Article. http://doi.org/10.1111/j.1369-7625.2004.00299.x

Chaudoir, S., Dugan, A., & Barr, C. H. I. (2013). Measuring factors affecting implementation of health innovations: a systematic review of structural, organizational, provider, patient, and innovation level measures. *Implementation Science*, *8*(1), 22. Journal Article. Retrieved from http://www.implementationscience.com/content/8/1/22

Colquhoun, H. L., Levac, D., O'Brien, K. K., Straus, S., Tricco, A. C., & Perrier, L. (2014). Scoping reviews: time for clarity in definition, methods, and reporting. *J Clin Epidemiol*, *67*. article. http://doi.org/10.1016/j.jclinepi.2014.03.013

Coulter, A. (2009). *Implementing shared decision making in the UK: A report for the Health Foundation*. Book, London: Health Foundation.

Damschroder, L. J., Aron, D. C., Keith, R. E., Kirsh, S. R., Alexander, J. A., & Lowery, J. C. (2009). Fostering implementation of health services research findings into practice: A consolidated framework for advancing implementation science. *Implementation Science*, *4*, 50. Journal Article. http://doi.org/10.1186/1748-5908-4-50

Daudt, H. M. L., van Mossel, C., & Scott, S. J. (2013). Enhancing the scoping study methodology: A large, inter-professional team's experience with Arksey and O'Malley's framework. *BMC Medical Research Methodology*, *13*(48). Journal Article.

Elwyn, G., Frosch, D.L., & Kobrin, S. (2016). Implementing shared decision-making: consider all the consequences. I*mplementation Science, 11*, 114.

Elwyn, G., Laitner, S., Coulter, A., Walker, E., Watson, P., & Thomson, R. (2010). Implementing shared decision making in the NHS. *British Medical Journal*, *341*, 971–975. Journal Article. http://doi.org/10.1136/bmj.c5146

Foundation for Informed Medical Decision Making. (2009). *Informing and involving patients in medical decisions: The primary care physicians' perspective. Findings from a national survey of physicians*. Book, Boston.

Khalil, H., Peters, M., Godfrey, C. M., Mcinerney, P., Soares, C. B., & Parker, D. (2016). An Evidence-Based Approach to Scoping Reviews. *Worldviews on Evidence-Based Nursing*, *13*(2), 118–123. http://doi.org/10.1111/wvn.12144

Légaré, F., Ratté, S., Gravel, K., & Graham, I. D. (2008). Barriers and facilitators to implementing shared decision-making in clinical practice: Update of a systematic review of health professionals' perceptions. *Patient Education and Counseling*, *73*(3), 526–535. Jhttp://www.ncbi.nlm.nih.gov/entrez/query.fcgi?cmd=Retrieve&db=PubMed&dopt=Citation&list_uids=18752915

Levac, D., Colquhoun, H., & O'Brien, K. K. (2010). Scoping studies: advancing the methodology. *Implementation Science*, *5*(69). Journal Article.

Moher, D., Shamseer, L., Clarke, M., Ghersi, D., Liberati, A., Petticrew, M., … Stewart, L. A.

(2015). Preferred Reporting Items for Systematic Review and Meta-Analysis Protocols (PRISMA-P) 2015 statement. *Systematic Reviews*, *4*(1), 1. http://doi.org/10.1186/2046-4053-4-1

Müller, E., Hahlweg, P., & Scholl, I. (2016). What do different stakeholders need to better implement shared decision making in cancer care? A qualitative needs assessment. *Acta Oncologica*, (September), 1–8. http://doi.org/10.1080/0284186X.2016.1227087

Shepherd, H. L., Tattersall, M. H., & Butow, P. N. (2007). The context influences doctors' support of shared decision-making in cancer care. *British Journal of Cancer*, *97*(1), 6–13. Journal Article. Retrieved from http://www.ncbi.nlm.nih.gov/entrez/query.fcgi?cmd=Retrieve&db=PubMed&dopt=Citation&list_uids=17551491

Shepherd, H. L., Tattersall, M. H., & Butow, P. N. (2008). Physician-identified factors affecting patient participation in reaching treatment decisions. *Journal of Clinical Oncology*, *26*(10), 1724–1731. Journal Article. http://doi.org/10.1200/jco.2007.13.5566

Stiggelbout, A. M., van der Weijden, T., de Wit, M. P. T., Frosch, D., Légaré, F., Montori, V. M., … Elwyn, G. (2012). Shared decision making: Really putting patients at the centre of healthcare. *British Medical Journal*, *344*, e256. Journal Article. http://doi.org/10.1136/bmj.e256

Vogel, B. A., Helmes, A. W., & Hasenburg, A. (2008). Concordance between patients' desired and actual decision-making roles in breast cancer care. *Psychooncology*, *17*(2), 182–189. Journal Article. http://doi.org/10.1002/pon.1215
