# Supplementary material for: Organizational- and system-level characteristics that influence implementation of shared decision-making and strategies to address them — a scoping review
Source: Implement Sci. 2018 Mar 9;13:40. doi: 10.1186/s13012-018-0731-z (PMC5845212; doi:10.1186/s13012-018-0731-z)
Supplement: Supplementary file 2 — Electronic searches. (PDF 24 kb) [file 13012_2018_731_MOESM2_ESM.pdf]

PubMed, WoS and CINAHL searches run on Oct 10, 2016

	Search results	Imported into EndNote	Imported into EndNote duplicates library (automatically)	Duplicates found manually (IS)
PubMed	5216	5216	0	16
Web of Science	5047	2806	2241	773
CINAHL	506	431	75	40
Totals	10,679	8363	2316	829

PubMed

(("Decision Making"[Mesh] AND ("patient participation" [mesh] OR "patient-centered care" [mesh] OR "patient preference"[mesh])) OR ("Decision support techniques" [mesh] AND ("patient participation" [mesh] OR "patient-centered care" [mesh] OR "patient preference"[mesh])) OR Shared decision making [tiab] OR Decision support [tiab] OR Patient engagement [tiab] OR Decision aid* [tiab] OR Patient involvement [tiab] OR Informed decision* [tiab]) AND ("Program Evaluation"[Mesh] OR "Organizational Culture"[mesh] OR "Quality Improvement"[Mesh] OR Program evaluation [tiab] OR Organizational culture [tiab] OR Organisational culture [tiab] OR Quality improvement [tiab] OR Implement* [tiab] OR Routine care [tiab] OR Routine practice [tiab] OR Routine clinical practice [tiab] OR Knowledge translation [tiab])

Filters activated: Publication date from 1997/01/01, English, German.

Search run Oct 10, 2016 = 5216 results

CINAHL

(((MH "Decision Making+" AND (MH "Consumer Participation" OR MH "Patient Centered Care" OR (MH "Patient Education")) OR (MH "Decision Support Techniques+" AND (MH "Consumer Participation" OR MH "Patient Centered Care" OR (MH "Patient Education")) OR "Shared decision making" OR "Decision support" OR "Patient engagement" OR "Decision aid*" OR "Patient involvement" OR "Informed decision*"))

AND

((MH "Program Evaluation" OR MH "Organizational Culture" OR MH "Quality Improvement+" OR "Program evaluation" OR "Organizational culture" OR "Organisational culture" OR "Quality improvement" OR Implement* OR "Routine care" OR "Routine practice" OR "Routine clinical practice" OR "Knowledge translation"))

Date limit: 1997-present

Exclude MEDLINE records

Limit to English or German: 506 hits (search run Oct 10, 2016)

Web of Science

("Shared decision making" OR "Decision support" OR "Patient engagement" OR "Decision aid*" OR "Patient involvement" OR "Informed decision*") AND ("Program evaluation" OR "Organizational culture" OR "Organisational culture" OR "Quality improvement" OR Implement* OR "Routine care" OR "Routine practice" OR "Routine clinical practice" OR "Knowledge translation") AND (Medicine OR Medical OR Hospital* OR Nurs* OR Health OR patient* OR clinical)

Date limit: 1997-present
Language: German or English
Oct 10, 2016 search: 5047 results
